# Downregulation of ATM and BRCA1 Predicts Poor Outcome in Head and Neck Cancer: Implications for ATM-Targeted Therapy

**DOI:** 10.3390/jpm11050389

**Published:** 2021-05-10

**Authors:** Yu-Chu Wang, Ka-Wo Lee, Yi-Shan Tsai, Hsing-Han Lu, Si-Yun Chen, Hsin-Ying Hsieh, Chang-Shen Lin

**Affiliations:** 1 Graduate Institute of Medicine, College of Medicine, Kaohsiung Medical University, Kaohsiung 807, Taiwan; ycwang0214@gmail.com (Y.-C.W.); 1016ys@gmail.com (Y.-S.T.); unrealhank@gmail.com (H.-H.L.); qoxoruby@gmail.com (S.-Y.C.); hsinying11125@gmail.com (H.-Y.H.); 2 Department of Otorhinolaryngology, Kaohsiung Municipal Ta-Tung Hospital, Kaohsiung 801, Taiwan; kawolee@kmu.edu.tw; 3 Hepatobiliary Division, Department of Internal Medicine, Kaohsiung Medical University Hospital, Kaohsiung Medical University, Kaohsiung 807, Taiwan; 4 Center for Cancer Research, Kaohsiung Medical University, Kaohsiung 807, Taiwan; 5 Department of Medical Research, Kaohsiung Medical University Hospital, Kaohsiung Medical University, Kaohsiung 807, Taiwan; 6 Department of Biological Sciences, National Sun Yat-Sen University, Kaohsiung 804, Taiwan

**Keywords:** *ATM*, *BRCA1*, biomarker, betel quid, head and neck cancer, KU55933, prognosis

## Abstract

*ATM* and *BRCA1* are DNA repair genes that play a central role in homologous recombination repair. Alterations of *ATM* and *BRCA1* gene expression are found in cancers, some of which are correlated with treatment response and patient outcome. However, the role of *ATM* and *BRCA1* gene expression in head and neck cancer (HNC) is not well characterized. Here, we examined the prognostic role of *ATM* and *BRCA1* expression in two HNC cohorts with and without betel quid (BQ) exposure. The results showed that the expression of *ATM* and *BRCA1* was downregulated in BQ-associated HNC, as the BQ ingredient arecoline could suppress the expression of both genes. Low expression of either *ATM* or *BRCA1* was correlated with poor overall survival (OS) and was an independent prognostic factor in multivariate analysis (*ATM* HR: 1.895, *p* = 0.041; *BRCA1* HR: 2.163, *p* = 0.040). The combination of *ATM* and *BRCA1* expression states further improved on the prediction of OS (HR: 4.195, *p* = 0.001, both low vs. both high expression). Transcriptomic analysis showed that inhibition of ATM kinase by KU55933 induced apoptosis signaling and potentiated cisplatin-induced cytotoxicity. These data unveil poor prognosis in the HNC patient subgroup with low expression of *ATM* and *BRCA1* and support the notion of *ATM*-targeted therapy.

## 1. Introduction

DNA repair genes function as an anti-cancer barrier in the early stage of tumorigenesis [1,2]. In response to DNA damage, the ataxia telangiectasia mutated (ATM) kinase quickly phosphorylates histone H2A.X (γ-H2AX) and downstream targets, such as p53, BRCA1, and CHEK1/2, resulting in activation of the DNA damage response (DDR) that halts the cell cycle and initiates DNA repair process [3]. In addition to the role in DNA repair, *BRCA1* is involved in the control of gene expression, cell cycle checkpoint, and centrosome duplication to keep genome integrity [4,5,6]. Thus, the normal functions of *ATM* and *BRCA1* are required for preventing malignant transformation, and defects in *ATM* and *BRCA1* genes are observed in human cancers [7,8,9,10,11,12].

Nevertheless, DDR also plays a critical role for cancer cells in response to anti-cancer therapy [13,14], which influences cancer patient outcomes. For example, cancer cells with low expression of *BRCA1* are sensitive to DNA-damaging agents, such as cisplatin, but are resistant to spindle poisons, such as taxol [15,16]. As a result, the expression of *BRCA1* can serve as a prognostic biomarker in breast, ovarian, lung, and colorectal cancers [10,17,18,19]. *ATM*-deficient cells are susceptible to ionizing radiation [20]; however, loss of one *ATM* allele or low ATM expression confers radioresistance to head and neck cancer (HNC) cells [12]. In this regard, we found that laryngeal and pharyngeal cancer patients with low *ATM* expression exhibited a poor overall survival (OS) rate [8]. Low *ATM* expression is also correlated with poor survival in uveal melanoma, breast and gastric cancers [21,22,23]. These studies indicate that the expression of *ATM* and *BRCA1* in cancer cells can modulate therapeutic efficacy and patient outcome.

In addition to serving as a biomarker, *ATM* and *BRCA1* are potential targets for anti-cancer therapy. Although not a direct drug target, cancer patients with *BRCA1* mutation or homologous recombination deficiency (HRD) exhibit a good response to the treatment with PARP inhibitors, such as olaparib [24]. HRD can also be caused by defects in the genes involved in homologous recombination repair (HRR), such as *ATM*. Nowadays, regardless of histological types and anatomic sites, a lot of basket trials are ongoing to evaluate the efficacy of PARP inhibitors on the cancer patients with HRD phenotype, including *ATM*-deficient cancers and HNC [24,25,26,27,28]. Because ATM kinase plays a central role in initiating DDR, targeting ATM kinase may improve the efficacy of DNA-damaging-based treatment by suppressing DNA repair in cancer cells. Some selective ATM kinase inhibitors have been developed and evaluated in preclinical studies as well as in clinical trials [29,30].

HNC includes the malignancy in larynx, pharynx, oral and nasal cavities. The treatment option for HNC, depending on disease status, includes surgical resection, chemotherapy (cisplatin, 5-fluorouracil, paclitaxel), radiotherapy, target therapy (cetuximab), and immune therapy (pembrolizumab, nivolumab) [31]. In spite of these treatments, the 5-year survival rate of HNC patients remains to have no significant improvement across the past decades. Therefore, there is an urgent need to develop new therapeutics for better treatment of HNC patients. To this end, a useful biomarker is required for an appropriate choice of such therapeutics. Because HNC is heterogeneous, an ideal molecular biomarker across different anatomic subsites is a warrant of better stratification and treatment of HNC.

Previously, we have reported an association between low *ATM* expression and poor patient outcome in laryngeal and pharyngeal cancers [8]. Yu et al. also found that low ATM expression was correlated with a worse OS in oral cancer [32]. The HNC patients both in Yu et al.’s and our studies have a habit of chewing betel quid (BQ), which ingredients may suppress the expression of *ATM* and *BRCA1* [32,33]. The promoting effect of BQ on the development of HNC has been demonstrated by epidemiological investigations [34,35]. We have also illustrated that the major alkaloid of BQ, arecoline, exerts its carcinogenic activity through inhibiting DNA repair and deregulating chromosome segregation [36,37,38,39]. Thus, in addition to cigarette smoking, alcohol drinking, and human papillomavirus infection as those common etiology in the Western countries, BQ-chewing is a risk factor for HNC in BQ-epidemic regions. However, the difference in HNC molecular characteristics between the Western countries and BQ-epidemic areas is unclear, which may hamper a generalized application of new therapeutics to both patient groups.

In this study, we confirmed the inhibitory effect of BQ and its ingredient arecoline on the expression of *ATM* and *BRCA1*, and compared the prognostic roles of *ATM* and *BRCA1* in HNC with and without BQ exposure. We also demonstrated that inhibition of ATM kinase induced apoptosis signaling and potentiated cisplatin-induced cytotoxicity in cancer cells, which implies a potential treatment strategy for the HNC patients with low *ATM* and *BRCA1* expression.

## 2. Materials and Methods

### 2.1. Cell Culture and Chemicals

The human squamous cell carcinoma (SCC, HEp-2 and KB) and oral cancer (CAL27 and SAS) cell lines were cultured in Dulbecco’s modified Eagle’s medium (HyClone, Logan, UT, USA) and 10% fetal bovine serum (Invitrogen, Carlsbad, CA, USA) at 37 °C and 5% CO_2_ [36,40]. These cells were periodically checked for free of mycoplasma using EZ-PCR Mycoplasma Test kit (Biological Industries, Israel). In case of contamination, cells were treated with Plasmocure (Invivogen, San Diego, CA, USA) to eliminate mycoplasma infection. Arecoline and cisplatin were purchased from Sigma (St. Louis, MO, USA). The selective ATM kinase inhibitor KU55933 was obtained from BioVision (Mountain View, CA, USA). For arecoline treatment, an average concentration (0.3 mM) [41] found in the oral cavity of BQ chewers was used.

### 2.2. RNA Purification, Reverse Transcription, and Real-Time Quantitative PCR (RT-qPCR)

RNA purification and reverse transcription were performed using Tri-reagent (Sigma) and High-Capacity cDNA Archive Kit (Applied Biosystems, Foster City, CA), and then qPCR was conducted with PowerSYBR Green reagent and the StepOne System (Applied Biosystems) as described [8,36]. The qPCR condition was: 50 °C for 2 min, 95 °C for 10 min, and 40 cycles at 95 °C for 15 s, followed by 60 °C for 1 min and a dissociation (melting) curve analysis to verify the specificity of qPCR products. Each sample was run for target genes and *GAPDH*, which served as an internal control. The relative fold expression was calculated using the 2^−ΔΔC^_T_ method. The primer sequences are shown in Table 1.

### 2.3. Chromatin Immunoprecipitation and qPCR (ChIP-qPCR) Assay

ChIP-qPCR assay was performed by SimpleChIP Enzymatic Chromatin IP Kit (#9003, Cell Signaling, Danvers, MA, USA) as described [39]. Briefly, arecoline (0.3 mM, 24 h)-treated cells were fixed (1% formaldehyde, 10 min), neutralized (1X glycine, 5 min), and then sonicated using Q700 sonicator (Qsonica, Newtown, CT, USA). Immunoprecipitation was performed using anti-RNA polymerase II antibody (sc-899X, Santa Cruz, Santa Cruz, CA, USA) or normal immunoglobulin G (sc-2025, Santa Cruz, negative control) together with fragmented chromatin (5 μg) at 4 °C, overnight, and then the immunoprecipitated DNA was purified for qPCR. The PCR primer sequences were used for the proximal promoter regions of *ATM*: GTCGTCACTTCCGTCCTCAG and CCAGCGACTTAGCGTTTGC and of *BRCA1*: AAAGAGCCAAGCGTCTCTCG and CTTTCCTTTTACGTCATCCGGG.

### 2.4. Construction of the ATM Promoter-Luciferase Reporter and Dual Luciferase Assay

The *ATM* promoter (−532 to +55) was PCR amplified from the genomic DNA of HEp-2 cells using the primers: TGATCAAAACCACAGCAGGA and CTCTCGCCTCCTCCCGTG and was cloned into the pGEM-T vector. Subsequently, the *ATM* promoter region was restricted from the pGEM-T vector using SacII (repaired to blunt end) and SacI, and then was cloned into the SmaI and SacI sites of the pGL3-Basic luciferase reporter (Progema, Ipswich, WI, USA) to obtain pATM-Luc. The cloned *ATM* promoter sequence was confirmed by DNA sequencing. The pATM-Luc (250 ng) was co-transfected with pRL-CMV (30 ng, internal control) into HEp-2 cells using Lipofectamine 2000 (Invitrogen), then the cells were treated with various doses of arecoline for 24 h and the expressed luciferase was determined by dual-luciferase assay according to manufacturer’s instructions (Promega).

### 2.5. Analysis of ATM and BRCA1 mRNA Expression in BQ-associated HNC Specimens

The collection and analysis of HNC specimens were approved by the Institutional Review Board of Kaohsiung Medical University Hospital (KMUH, IRB-950094). All specimens were acquired after having the signed informed consents. In total, 100 HNC and 52 adjacent non-tumor tissues were analyzed. Among the 100 HNC, *ATM* expression in 36 laryngeal and 33 pharyngeal cancers have been reported in our previous paper [8]; the remaining 31 cases are oral cancers. There are 53 patients with treatment record of radiotherapy. The patient demographics is shown in Supplementary Table S1. The analysis of gene expression for each HNC and adjacent non-tumor sample was as the protocols described previously [8]. Each sample was examined by RT-qPCR at least for twice and an average value was used. For the HNC and non-tumor pairs, fold-expression was interpreted by a ratio of gene expression in HNC versus that in the paired non-tumor tissue. For the 48 HNC specimens without paired non-tumor tissues, we used the average expression of all non-tumor tissues as a reference for comparison.

### 2.6. Analysis of ATM and BRCA1 mRNA Expression in the Cancer Genome Atlas (TCGA)

The mRNA expression of *ATM* and *BRCA1* in TCGA HNSC cohort (for Figure 2C,D) was interpreted using the web-based analysis tool GEPIA2 (http://gepia2.cancer-pku.cn/#index) [42].

### 2.7. Transcriptome and Gene Set Enrichment Analysis (GSEA)

The HEp-2 cells were treated with KU55933 (10 μM) and DMSO for 24 h and then total RNA was purified using Tri-reagent (Sigma) for microarray analysis using the Human OneArray Plus platform (Phalanx Biotech, Taiwan) with array version: HOA 7.1. The raw data were processed using Rosetta Resolver system (Rosetta Biosoftware, Seattle, WA, USA). The resulted microarray data were applied for gene set enrichment analysis using GSEA version 4.03 and the gene set collections of hallmark, curated, and GO in the Molecular Signatures Database version 7.1 [43].

### 2.8. Western Blot Analysis

Western blot analysis was performed as described in our papers [36,39]. Briefly, cells were treated with KU55933 (20 μM) for indicated time periods and then were harvested using RIPA lysis buffer. Protein lysates (30 μg) were separated by sodium dodecyl sulfate-polyacrylamide gel electrophoresis, transferred to polyvinylidene difluoride membranes, detected antibodies, and then visualized by enhanced chemiluminescence (Millipore, Bedford, MA) and ChemiDoc-It imaging system (UVP, Upland, CA, USA). The primary antibodies CHK2 (sc-9064), BAX (sc-7480), PARP (sc-8007), and GAPDH (sc-32233) were purchased from Santa Cruz. The antibodies for the phosphorylated forms of ATM (#4526) and CHK2 (#2661) and cleaved caspase 3 (#9664) was obtained from Cell Signaling. The ATM antibody (Y170) was from Epitomics (Burlingame, CA, USA). The signal of GAPDH was used as an internal control.

### 2.9. Cell Viability and Caspase 3/7 Activity Assays

Cell viability was examined using 3-[4, 5-dimethylthiazol-2-yl]-2,5-diphenyltetrazolium bromide (MTT) (Sigma) assays as described [40]. Briefly, 5 × 10^3^ cells were seeded in each well of a 96-well plate one day before treatment by cisplatin (2 μM) and KU55933 (10 μM), either alone or in combination, and incubated for 48 h. Thereafter, MTT (125 μg/mL) was added for 1 h and the absorbance of DMSO-extracted purple formazans was measured at 540 nm using the VERSAmax microplate reader (Molecular Devices, Sunnyvale, CA, USA). For the measurement of caspase 3/7 activity, cells were treated with cisplatin (5 μM) and KU55933 (20 μM), either alone or in combination, for 24 h, and then the caspase 3/7 activity was determined by the Caspase-Glo 3/7 activity assay kit (Promega) according to manufacturer’s instruction.

### 2.10. Statistical Analysis

For all in vitro experiments, data from independent experiments (each with two replicates) were shown as mean ± standard deviation. The difference between two experiment groups was examined using Student’s *t*-test, *p* < 0.05 was considered significant. The IBM SPSS Statistics (version 22, Armonk, NY, USA) was used for clinical analysis; the correlation between categories was analyzed using chi square or Fisher’s exact test. The patient’s overall survival in the KMUH cohort was calculated by Kaplan-Meier estimates and the Log-rank tests. The hazard ratio was computed using multivariate Cox model. The patient’s overall survival in TCGA HNSC cohort was analyzed by the web-based tools Kaplan Meier plotter (for Figure 3E,F) (http://kmplot.com/analysis/) [44] and GEPIA2 (for Figure 4B, Supplementary Figure S1).

## 3. Results

### 3.1. Areca Nut Extract (ANE) and Arecoline Inhibit the Expression of ATM and BRCA1

**Figure 1:**
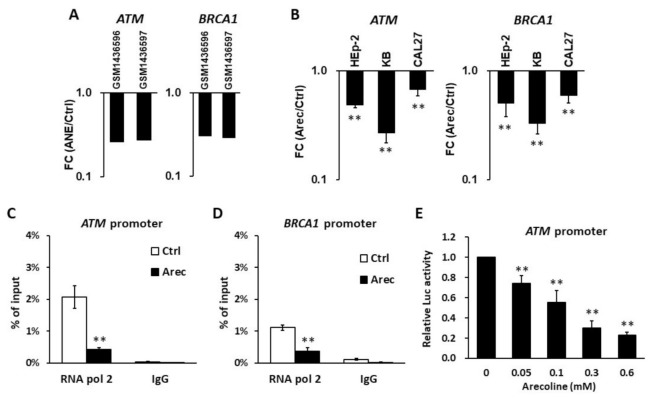
Areca nut extract (ANE) and arecoline inhibit the expression of *ATM* and *BRCA1*. (**A**) The biological duplicated data (GSM1436696 and GSM1436697) of *ATM* and *BRCA1* mRNA expression in ANE (5 μg/mL, 72 h)-treated hGF cells were extracted from the Gene Expression Omnibus (GSE59414) [45] and were log-transformed. FC, fold-changed (ANE versus H_2_O control). (**B**) RT-qPCR showed that the *ATM* and *BRCA1* mRNA levels were downregulated by arecoline treatment (0.3 mM, 24 h) in HEp-2, KB, and CAL27 cells. The expression of *ATM* and *BRCA1* in vehicle control (H_2_O) was set as one by using GAPDH as an internal control. (**C**,**D**) Chromatin immunoprecipitation assays showed that the recruitments of RNA polymerase II to the promoters of *ATM* (**C**) and *BRCA1* (**D**) were decreased in arecoline (0.3 mM, 24 h)-treated HEp-2 cells. (**E**) The luciferase reporter assay showed that arecoline inhibited the promoter activity of *ATM* in HEp-2 cells in a dose-dependent manner. All data (**B–E**) are shown as mean ± standard deviation (*n* = 3–5). Ctrl, vehicle control (H_2_O); Arec, arecoline; ** *p* < 0.01 versus control.

To explore the effects of ANE and arecoline on the expression of *ATM* and *BRCA1*, we first extracted the microarray data of ANE (5 μg/mL, 72 h)-treated human gingival fibroblasts (hGFs) from the Gene Expression Omnibus (GEO, GSE59414) [45] and found that both *ATM* and *BRCA1* mRNA expressions were downregulated by ANE treatment (Figure 1A). Next, we applied RT-qPCR to examine the effect of arecoline, the principal alkaloid of ANE, on the expression of *ATM* and *BRCA1* in cancer cells. The results showed that arecoline suppressed the expression of *ATM* and *BRCA1* mRNAs in HEp-2, KB, and CAL27 SCC cells (Figure 1B). Chromatin immunoprecipitation and qPCR assay confirmed that the binding of RNA polymerase II to the promoters of *ATM* (Figure 1C) and *BRCA1* (Figure 1D) was decreased upon arecoline treatment. The promoter of *ATM* was cloned into a luciferase reporter (pATM-Luc) and then was transfected into HEp-2 cells with arecoline treatment. The results showed that arecoline was able to inhibit *ATM* promoter activity in a dose-dependent manner (Figure 1E). Tu et al. and our unpublished data also showed that arecoline could decrease *ATM* promoter activity in SAS, OEC-M1 [32], and KB cells (data not shown). These results indicate that ANE and arecoline suppress the expression of *ATM* and *BRCA1* in hGFs and cancer cells.

### 3.2. ATM and BRCA1 Are Downregulated in HNC and Are Correlated with Poor Patient Outcome

**Figure 2:**
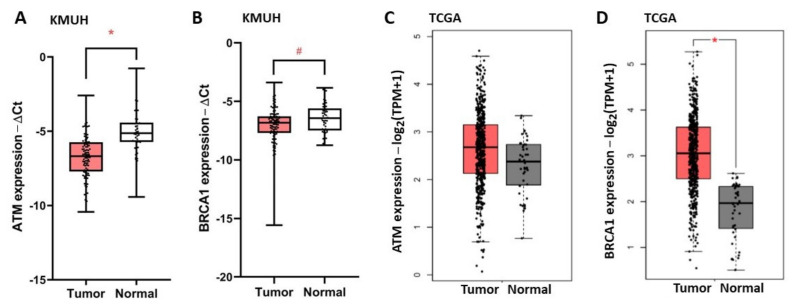
The expressions of *ATM* and *BRCA1* mRNA are downregulated in BQ-associated HNC. (**A**,**B**) The relative expression of *ATM* (**A**) and *BRCA1* (**B**) mRNAs in HNC (*n* = 100) versus adjacent non-tumor (normal) tissues (*n* = 52) of the Kaohsiung Medical University Hospital (KMUH) cohort were shown as box plots. (**C**,**D**) The expression of *ATM* (**C**) and *BRCA1* (**D**) mRNAs in HNC (*n* = 519) and in adjacent normal tissues (*n* = 44) of The Cancer Genome Atlas (TCGA) cohort were shown as box plots. TPM, transcripts per million. # *p* < 0.05; * *p* < 0.01 (Mann-Whitney U test).

To investigate the role of *ATM* and *BRCA1* in HNC, we examined the expression of *ATM* and *BRCA1* in 100 BQ-associated HNC specimens, named as the Kaohsiung Medical University Hospital (KMUH) cohort, using RT-qPCR. The patient demographics are shown in the Supplementary Table S1. Figure 2 shows the relative expression of *ATM* and *BRCA1* in tumor versus adjacent non-tumor tissues. The expression of *ATM* (Figure 2A) and *BRCA1* (Figure 2B) was downregulated (tumor/non-tumor < 1) in 80 and 68 cases of HNC, respectively. In contrast, the expression of *ATM* (Figure 2C) and *BRCA1* (Figure 2D) was not decreased in the HNC specimens of The Cancer Genome Atlas (TCGA) cohort, which did not expose to BQ.

**Figure 3:**
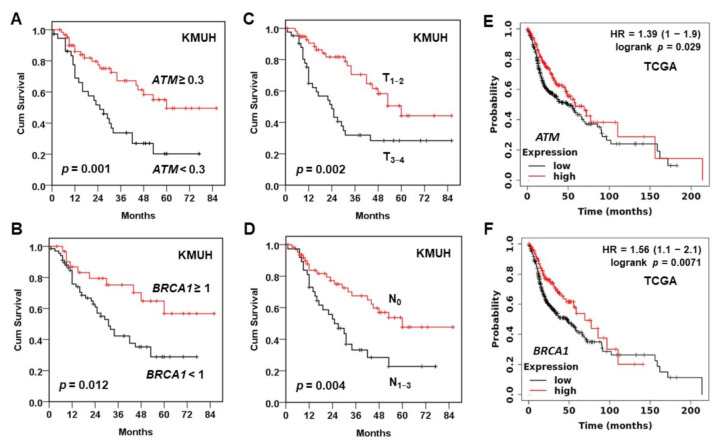
Kaplan-Meier analysis of overall survival (OS). OS curves for (**A**) *ATM* (<0.3, *n* = 37; >0.3, *n* = 63), (**B**) *BRCA1* (<1, *n* = 68; >1, *n* = 32), (**C**) tumor size (T_1–2_, *n* = 58; T_3–4_, *n* = 42), and (**D**) lymph node involvement (N_0_, *n* = 63; N_1–3_, *n* = 37) in the KMUH cohort. (**E**,**F**) OS curves for (**E**) *ATM* (low, *n* = 334; high, *n* = 165) and (**F**) *BRCA1* (low, *n* = 352; high, *n* = 147) in TCGA cohort were analyzed using Kaplan Meier plotter (http://kmplot.com/analysis/) with the mode of “auto select best cutoff”.

The cutoffs of *ATM* (0.3) and *BRCA1* (1.0) expressions (tumor/non-tumor) were determined by receiver operating characteristic analysis. The downregulation of *ATM* and *BRCA1* mRNA was correlated with worse overall survival (OS) of HNC patients in the KMUH cohort (Table 2). Kaplan-Meier survival analysis (Figure 3, Table 3) demonstrated shorter 5-yr OS rates in the patients with downregulated *ATM* (20.2% vs. 49.5%, *p* = 0.001) or *BRCA1* (28.9% vs. 56.7%, *p* = 0.012). Patients with large tumor size (28.3% vs. 44.2%, *p* = 0.002) and lymph node involvement (22.8% vs. 47.7%, *p* = 0.004) exhibited shorter 5-yr OS rates (Table 3). A worse OS was also observed in the HNC patients of TCGA cohort who exhibited a low expression of *ATM* (Figure 3E) or *BRCA1* (Figure 3F), especially in the patients with atypical and classic molecular subtypes [46] (Supplementary Figure S1). Multivariate Cox model analysis showed that large tumor size (HR = 2.046, 95% CI: 1.116–3.752), positive lymph node (HR = 2.085, 95% CI: 1.096–3.966), downregulated expression of *ATM* (HR = 1.895, 95% CI: 1.026–3.501) and *BRCA1* (HR = 2.163, 95% CI: 1.037–4.511) were independent poor prognostic factors for HNC patients in the KMUH cohort (Table 4).

### 3.3. Combination of Two Prognosis Factors Improves Outcome Prediction

**Figure 4:**
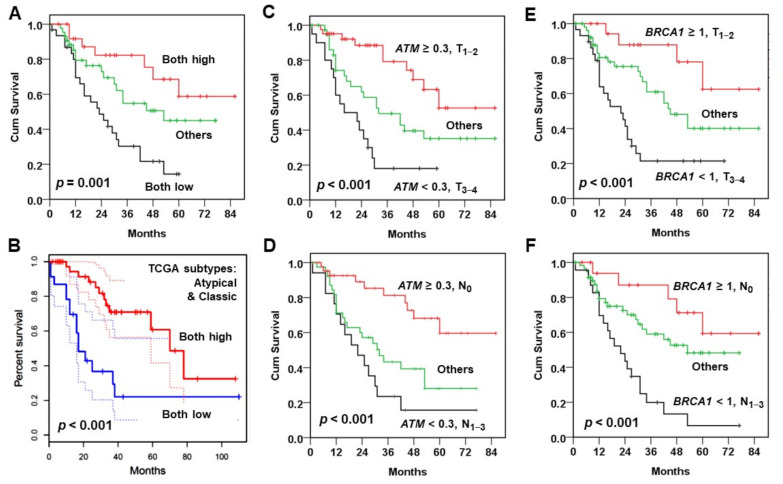
Kaplan-Meier analysis of overall survival (OS) with the combination of two prognosis factors. (**A**,**B**) OS curves for the combination of *ATM* and *BRCA1* in (**A**) the KMUH cohort (*n* for both high: 26, both low: 31, others: 43) and in (**B**) TCGA cohort (atypical and classic molecular subtypes, *n* for both high: 41, both low: 23). (**C**,**D**) OS curves for the combination of *ATM* and (**C**) tumor size (*n* for *ATM* > 0.3 and T_1–2_: 41, *ATM* < 0.3 and T_3–4_: 20, others: 39) or (**D**) lymph node involvement (*n* for *ATM* > 0.3 and N_0_: 43, *ATM* < 0.3 and N_1–3_: 17, others: 40) in the KMUH cohort. (**E**,**F**) OS curves for the combination of *BRCA1* and (**E**) tumor size (*n* for *BRCA1* > 1 and T_1–2_: 19, *BRCA1* < 1 and T_3–4_: 29, others: 52) or (**F**) lymph node involvement (*n* for *BRCA1* > 1 and N_0_: 18, *BRCA1* < 1 and N_1–3_: 23, others: 59) in the KMUH cohort.

The prediction of patient OS was improved when combined with two independent prognostic factors. In the KMUH cohort, the patients simultaneously with low *ATM* and *BRCA1* mRNA levels had the poorest OS; in contrast, the ones with both high expression of *ATM* and *BRCA1* exhibited the best outcome (Figure 4A). Similarly, the patients in TCGA cohort with high expression of *ATM* and *BRCA1* showed better OS than those with low expression of both genes (Figure 4B). The patients (KMUH cohort) with two good prognostic factors, such as *ATM*–high expression and small tumor size (Figure 4C) or negative lymph node (Figure 4D), showed superior OS to other subgroups. The patients with *BRCA1*–high expression and small tumor size (Figure 4E) or negative lymph node (Figure 4F) also showed an improved OS. Multivariate Cox model analysis (Table 5) confirmed that the patients with two worse prognosis factors exhibited an increased risk of death, such as the patients with double decreased *ATM* and *BRCA1* mRNAs (HR = 4.195, 95% CI: 1.779–9.892), downregulated *ATM* and large tumor (HR = 5.519, 95% CI: 2.456–12.405) or positive lymph node (HR = 5.143, 95% CI: 2.202–12.015), and downregulated *BRCA1* and large tumor (HR = 6.517, 95% CI: 2.204–19.264) or positive lymph node (HR = 5.543, 95% CI: 2.005–15.326). These results suggest that patient stratification by more than one prognostic factor can improve the prediction of patient outcome.

### 3.4. Inhibition of ATM Kinase Induces Apoptosis in Cancer Cells

To improve the poor OS of HNC patients with low *ATM* expression, inhibition of ATM kinase activity may be a potential strategy. Parikh et al. have shown that loss of one *ATM* allele with decreased ATM expression confers radioresistance in HNC cells [12]. Thus, HNC patients with low *ATM* expression may have a reduced response to anti-cancer therapy. By contrast, the cells with complete loss of *ATM* gene, such as the cells derived from ataxia-telangiectasia patients, are susceptible to DNA-damaging agents [20], which may also be true for cancer cells. To examine this notion, we have found that the selective ATM kinase inhibitor KU55933 can inhibit DNA damage-induced *ATM* signaling (Supplementary Figure S2) and decrease cell viability of cancer cells [40].

**Figure 5:**
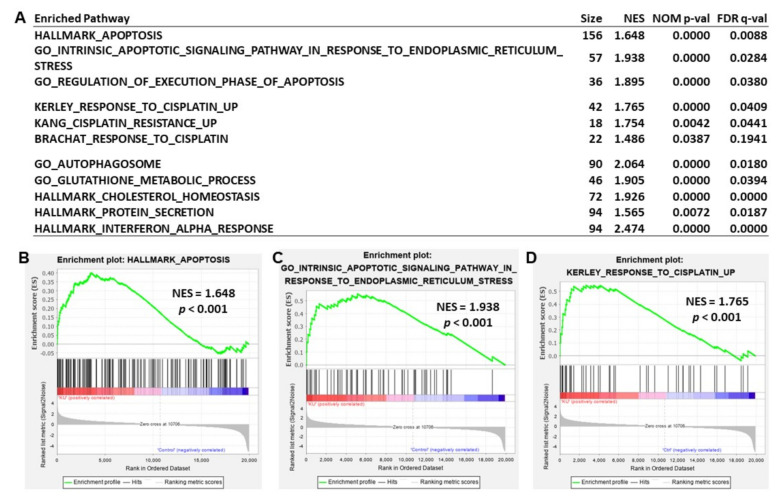
The ATM kinase inhibitor KU55933 induces apoptosis and other cellular pathways in HEp-2 cells. (**A**) Gene set enrichment analysis showed some representative pathways induced by KU55933. Additional KU55933-enriched pathways can be found in Supplementary Table S2. (**B**–**D**) Enrichment plots show the KU55933-enriched genes (left side) in the gene sets of HARLLMARK_APOPTOSIS (**B**), GO_APOPTOTIC_SIGNALING_PATHWAY_IN_ RESPONSE_TO_ENDOPLASMIC_RETICULUM STRESS (**C**), and KERLEY_RESPONSE_TO_ CISPLATIN_UP (**D**). The significant core enriched genes can be found in Supplementary Table S3. Size, gene number in the pathways. NES, normalized enrichment score. NOM, nominal. FDR, false discovery rate.

To explore the mechanism underlying KU55933-mediated suppression of cancer cell viability, we conducted a transcriptomic analysis in the KU55933-treated HEp-2 cells (10 μM for 24 h). The gene set enrichment analysis (GSEA) showed that KU55933 significantly induced several cellular pathways, such as apoptosis, response to cisplatin, autophagosome, glutathione metabolism, interferon response, and so on (Figure 5A). The significantly enriched pathways induced by KU55933 in the gene set collections of hallmark (top 20), curated (top 50), and GO (top 30) are shown in Supplementary Table S2. The representative enrichment plots for “apoptosis” gene sets in the hallmark and gene ontology (GO) collections are shown in Figure 5B,C, respectively. Figure 5D shows an enrichment plot for the “response to cisplatin” gene set. The significant core enriched genes in these three representative gene sets are list in Supplementary Table S3.

To confirm the effect of KU55933 on inducing apoptosis in cancer cells, a flow cytometric analysis for propidium iodide-staining of KU55933-treated HEp-2 cells was performed. A representative result shows that the KU55933 treatment increased the sub-G1 cell fraction from 1.3% to 11.1% (Supplementary Figure S3A). KU55933 treatment also increased the expression of BAX and the amounts of cleaved caspase 3 and PARP in HEp-2 and KB cells (Supplementary Figure S3B). These results indicate that KU55933 is able to induce apoptosis in cancer cells.

### 3.5. Inhibition of ATM Kinase Potentiates Cisplatin-Induced Cytotoxicity in Cancer Cells

**Figure 6:**
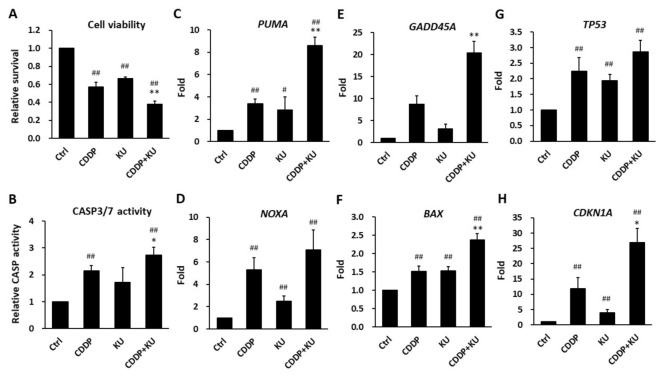
The ATM kinase inhibitor KU55933 potentiates cisplatin-induced cytotoxicity in HEp-2 cells. (**A**) Cell viability was decreased by the combination of cisplatin (2 μM) and KU55933 (10 μM) when compared with that by either cisplatin or KU55933 treatment alone for 48 h. (**B**) Caspase 3/7 activity was further increased by the combination treatment of cisplatin (5 μM) and KU55933 (20 μM) for 24 h. (**C**–**H**) RT-qPCR analysis for the gene expression of *PUMA* (**C**), *NOXA* (**D**), *GADD45A* (**E**), *BAX* (**F**), *TP53* (**G**), and *CDKN1A* (**H**) after treatments of cisplatin (5 μM) and KU55933 (10 μM), either alone or in combination, for 24 h. All data are shown as mean ± standard deviation (*n* = 3). Ctrl, vehicle control. CDDP, cisplatin. KU, KU55933. * *p* < 0.05 versus CDDP. ** *p* < 0.01 versus CDDP. # *p* < 0.05 versus Ctrl. ## *p* < 0.01 versus Ctrl.

In addition to apoptosis, the results of GSEA suggested that the gene sets related to cisplatin response were significantly enriched in KU55933-treated HEp-2 cells (Figure 5A,D). Therefore, we evaluated whether KU55933 could enhance cisplatin-induced cytotoxicity in HEp-2 cells. The results of the MTT assay demonstrated that cisplatin (2 μM) treatment reduced cell viability to ~55% of the control cells and further decreased to ~40% in combination with KU55933 (10 μM) (Figure 6A). Either cisplatin or KU55933 alone activated caspase 3/7 activities in HEp-2 (Figure 6B), KB, and SAS (Supplementary Figure S4) cells, which was further increased by the combination of both drugs (Figure 6B, Supplementary Figure S4). The results of RT-qPCR showed that combination treatment induced apoptosis gene expression in HEp-2 (Figure 6C–H), KB (Supplementary Figure S5), and SAS (Supplementary Figure S6) cells than that induced by the single agent alone, such as *PUMA* (8.6-fold by combination vs. 3.4- and 2.9-fold by cisplatin and KU55933, respectively), *NOXA* (7.1-fold vs. 5.3- and 2.5-fold, respectively), *GADD45A* (20.3-fold vs. 8.7- and 3.2-fold, respectively), *BAX* (2.4-fold vs. 1.5- and 1.5-fold, respectively), *TP53* (2.9-fold vs. 2.2- and 1.95-fold, respectively), and *CDKN1A* (26.9-fold vs. 11.8- and 4.0-fold, respectively) in HEp-2 cells. These results indicate that inhibition of ATM kinase by KU55933 augments cisplatin-induced cytotoxicity in cancer cells.

## 4. Discussion

*ATM* and *BRCA1* play a pivotal role in DNA repair, especially in the HRR pathway. In this study, we explore the prognostic role of *ATM* and *BRCA1* expression in HNC patients with and without BQ exposure, which are represented by the KMUH and TCGA cohorts, respectively. Downregulated *ATM* and *BRCA1* expressions were observed in the HNC patients of KMUH but not in TCGA cohort (Figure 2), possibly due to the inhibitory activity of the BQ ingredient arecoline on the expression of *ATM* and *BRCA1* (Figure 1).

In addition to the inhibitory activity of BQ/arecoline (Figure 1) [32,33], there are several mechanisms that may lead to downregulation of *ATM* and *BRCA1* in HNC. Hypermethylation of *ATM* promoter and loss of *ATM* gene at chromosome 11q22-23 are reported in HNC [47,48,49]. For *BRCA1*, both BQ/arecoline and loss of heterozygosity at chromosome 17q *BRCA1* locus have been reported [33,50,51]. These studies suggest that downregulation of *ATM* and *BRCA1* expression in HNC can be through multiple pathways.

Regardless of BQ exposure, it is true for both cohorts that the subgroup of HNC patients with lower expression of *ATM* and *BRCA1* exhibit a poor OS than those with higher expression of both genes (Figure 3). Either the downregulated *ATM* or *BRCA1* mRNA serves as an independent poor prognostic marker for BQ-associated HNC patients (Table 4). The combination of both markers further increases the prediction power on OS (Table 5). However, due to the limited patient number and treatment information in the present study, it is required to include more cases with treatment data to validate this association and to determine treatment responses to various therapeutics based on *ATM* and *BRCA1* expression in different HNC patient subgroups. Nevertheless, the present study sheds light on the importance of the clinical decision on treatment choice for HNC patients according to *ATM* and *BRCA1* and/or additional DDR gene expression.

It is well known that inhibition of *ATM* sensitizes cancers to genotoxic exposure. However, Park et al. have found that HNC cells derived from patients with loss of one *ATM* allele or low ATM expression are resistant to radiotherapy [12]. This finding is consistent with the present results that HNC patients with low *ATM* expression exhibit poor survival. Therefore, HNC patients with low *ATM* level may require therapeutics other than radiotherapy. In this regard, the present study demonstrates that direct targeting of ATM kinase by KU55933 induces apoptosis in cancer cells (Figure 5 and Figure 6, Supplementary Figures S3–S6). Several studies also show that KU55933 can increase apoptosis in combination with radiation or chemotherapeutic drugs [29,52,53,54]. Furthermore, our transcriptomic analysis suggests that KU55933 can modulate cellular responses to autophagy, cisplatin, glutathione and lipid metabolism, interferon, and many others. These pathways are essential for cancer cell growth and in response to various stresses, including that are induced by anti-cancer treatment and nutrition depletion. Of note, we have found that KU55933 activates interferon responsive genes, which may lead to tumors becoming “hot” and be vulnerable to immunotherapy. Of course, more investigations are needed to validate this notion. In addition to KU55933, other ATM kinase inhibitors, such as AZD0156, are developed and are shown to suppress cancer growth [55,56]. The clinical trial (NCT02588105) that aims to evaluate the efficacy of ATM kinase inhibitor is ongoing [30]. These studies support that *ATM*-targeted therapy is a potential strategy to improve the outcome of HNC patients with low *ATM* expression.

The present study finds an association between low expression of *ATM* and *BRCA1* and poor OS of HNC patients in the atypical and classic (Supplementary Figure S1, Figure 4B) but not in basal and mesenchymal (data not shown) molecular subtypes in TCGA cohort. The HNC in atypical subtype is associated with positive HPV infection [46]. Because HPV E6 and E7 oncoproteins can interact with BRCA1 [57], the effect of HPV-BRCA1 interaction on therapeutic response and survival in HPV-positive HNC patients is worthy to be examined in the future. The HNC in classic subtype exhibits a similar molecular feature as lung squamous cell carcinoma (LUSC); both are correlated with cigarette smoking [46]. Previous studies show that low *BRCA1* expression is correlated with poor outcome in LUSC patients but, in contrast, is associated with a better response to platinum-based chemotherapy in lung adenocarcinoma (LUAD) patients [58,59]. These results suggest that the poor prognostic role of low *BRCA1* expression may be restricted to SCC, such as LUSC and HNSC, but not in LUAD. The cross-talk between *ATM* and *BRCA1* expression and HPV and cigarette smoke on the influence of SCC patient outcome is an interesting question that can be investigated further.

## 5. Conclusions

This study demonstrates the value of *ATM* and *BRCA1* expression in the prediction of HNC patient survival. However, the relationship between *ATM* and *BRCA1* expression and the efficacies of various treatments in HNC patients needs to be evaluated in the future. Anti-cancer therapy based on the personalized *ATM* and *BRCA1* expression can probably improve the outcome of HNC patients. The ATM kinase inhibitor KU55933 is able to potentiate cisplatin-induced cytotoxicity, supporting the rationale of *ATM*-target therapy. The efficacy of ATM kinase inhibitor on HNC patients with different settings is worthy to be examined in the future.

## Figures and Tables

**Table 1:** Primer sequences for quantitative PCR.

Gene	Forward Primer (5′ to 3′)	Reverse Primer (5′ to 3′)
*ATM*	ATAGATTGTGTAGGTTCCGATGG	CATCTTGTCTCAGGTCATCACG
*BRCA1*	TTGTTGATGTGGAGGAGCAA	GATTCCAGGTAAGGGGTTCC
*PUMA*	GACCTCAACGCACAGTACGA	GAGATTGTACAGGACCCTCCA
*NOXA*	GGAGATGCCTGGGAAGAAG	CCTGAGTTGAGTAGCACACTCG
*BAX*	AGCAAACTGGTGCTCAAGG	TCTTGGATCCAGCCCAAC
*GADD45A*	TTTGCAATATGACTTTGGAGGA	CATCCCCCACCTTATCCAT
*TP53*	AGGCCTTGGAACTCAAGGAT	CCCTTTTTGGACTTCAGGTG
*CDKN1A*	TCACTGTCTTGTACCCTTGTGC	GGCGTTTGGAGTGGTAGAAA
*GAPDH*	AGCCACATCGCTCAGACAC	GCCCAATACGACCAAATCC

**Table 2:** Correlation between clinicopathological variables and mRNA expression.

Variables		*ATM* mRNA ^a^				*p* ^b^	*BRCA1* mRNA ^a^				*p* ^b^
		<0.3 (%)		>0.3 (%)			<1 (%)		>1 (%)		
Gender	Male	34	(36.2)	60	(63.8)	0.667 ^c^	65	(69.1	29	(30.9)	0.381 ^c^
	Female	3	(50.0)	3	(50.0)		3	(50.0)	3	(50.0)	
Age	<55	15	(34.9)	28	(65.1)	0.703	30	(69.8)	13	(30.2)	0.742
	>55	22	(38.6)	35	(61.4)		38	(66.7)	19	(33.3)	
T	1–2	17	(29.3)	41	(70.7)	0.061	39	(67.2)	19	(32.8)	0.848
	3–4	20	(47.6)	22	(52.4)		29	(69.0)	13	(31.0)	
N	0	20	(31.7)	43	(68.3)	0.156	45	(71.4)	18	(28.6)	0.338
	1–3	17	(45.9)	20	(54.1)		23	(62.2)	14	(37.8)	
Survival	No	26	(55.3)	21	(44.7)	<0.001	37	(78.7)	10	(21.3)	0.030
	Yes	11	(20.8)	42	(79.2)		31	(58.5)	22	(41.5)	

^a^ Ratio of tumor/normal. Cutoff determined by receiver operating characteristic analysis. ^b^ Chi-square test. ^c^ Fisher’s exact test.

**Table 3:** Kaplan-Meier analysis of overall survival rates.

Variables		3-yr (%)	5-yr (%)	*p* ^a^
Overall		53.1	37.4	
Gender	Male	53.0	36.7	0.657
	Female	53.3	-	
Age	<55	38.9	18.5	0.037
	>55	61.1	46.5	
T	1–2	31.9	28.3	0.002
	3–4	70.5	44.2	
N	0	33.2	22.8	0.004
	1–3	67.5	47.7	
*ATM* ^b^	<0.3	33.7	20.2	0.001
	>0.3	67.2	49.5	
*BRCA1* ^b^	<1	42.3	28.9	0.012
	>1	75.2	56.7	

^a^ Log-rank test. ^b^ Tumor/normal.

**Table 4:** Multivariate Cox model analysis of overall survival ^a^.

Variables		HR	95% CI	*p*
T	T_3–4_ vs. T_1–2_	2.046	1.116–3.752	0.021
N	N_1–3_ vs. N_0_	2.085	1.096–3.966	0.025
*ATM*	<0.3 vs. >0.3	1.895	1.026–3.501	0.041
*BRCA1*	<1 vs. >1	2.163	1.037–4.511	0.040

^a^ Adjusted by gender, age, T, N, *ATM*, and *BRCA1* mRNA expression.

**Table 5:** Multivariate Cox model analysis of overall survival with combined parameters ^a^.

Variables	HR	95% CI	*p*
*ATM*<0.3 & *BRCA1*<1	4.195	1.779–9.892	0.001
*ATM* > 0.3 & *BRCA1* < 1 or *ATM* < 0.3 & *BRCA1* > 1	1.815	0.747–4.406	0.188
*ATM* > 0.3 & *BRCA1* > 1	1		
*ATM* < 0.3 &T_3–4_	5.519	2.456–12.405	<0.001
*ATM* > 0.3 & T_3–4_ or *ATM* < 0.3 & T_1–2_	2.483	1.162–5.304	0.019
*ATM* > 0.3 & T_1–2_	1		
*ATM* < 0.3 & N_1–3_	5.143	2.202–12.015	<0.001
*ATM* > 0.3 & N_1–3_ or *ATM* < 0.3 & N_0_	3.001	1.406–6.407	0.005
*ATM* > 0.3 & N_0_	1		
*BRCA1* < 1 & T_3–4_	6.517	2.204–19.264	0.001
*BRCA1* > 1 & T_3–4_ or *BRCA1* < 1 & T_1–2_	2.839	0.965–8.358	0.058
*BRCA1* > 1 & T_1–2_	1		
*BRCA1* < 1 & N_1–3_	5.543	2.005–15.326	0.001
*BRCA1* > 1 & N_1–3_ or *BRCA1* < 1 & N_0_	1.963	0.738–5.220	0.177
*BRCA1* > 1 & N_0_	1		

^a^ Adjusted by gender, age.

## Data Availability

All data supporting reported results can be found through requesting the corresponding author. The TCGA HNSC dataset can be accessed at https://portal.gdc.cancer.gov.

## Supplementary Materials

The following are available online at https://www.mdpi.com/article/10.3390/jpm11050389/s1, Figure S1: Kaplan-Meier analysis of overall survival (OS) in the atypical and classic molecular subtypes in TCGA HNSC cohort, Figure S2: The ATM kinase inhibitor KU55933 inhibits camptothecin (CPT)- and cisplatin (CDDP)-induced ATM signaling in HEp-2 cells, Figure S3: The ATM kinase inhibitor KU55933 induces apoptosis in cancer cells, Figure S4: Caspase 3/7 activity is increased by the combination treatment of cisplatin (5 μM) and KU55933 (20 μM) for 24 h in KB and SAS cells, Figure S5: The ATM kinase inhibitor KU55933 augments cisplatin-induced apoptosis gene expression in KB cells, Figure S6: The ATM kinase inhibitor KU55933 augments cisplatin-induced apoptosis gene expression in SAS cells, Figure S7: The full-length blots for Figure S2B, Table S1: Patient demographics, Table S2: Significant enriched pathways, Table S3: Significant core enriched genes^a^ in the gene sets of HALLMARL_APOPTOSIS, GO_INTRINSIC_APOPTOTIC_SIGNALING_PATHWAY_IN_RESPONSE_TO_ENDOPLASMIC_RETICULUM_STRESS, and KERLEY_RESPONSE_TO_CISPLATIN_UP pathways.
