# Supplementary material for: Downregulation of ATM and BRCA1 Predicts Poor Outcome in Head and Neck Cancer: Implications for ATM-Targeted Therapy
Source: J Pers Med. 2021 May 10;11(5):389. doi: 10.3390/jpm11050389 (PMC8151497; doi:10.3390/jpm11050389)
Supplement: Supplementary file 1 [file jpm-11-00389-s001.zip › jpm-1198256-supplementary.pdf]

Supplementary Materials

Downregulation of ATM and BRCA1 predicts poor outcome in head and neck cancer: implications for ATM-targeted therapy

Yu-Chu Wang [1,†], Ka-Wo Lee [2,†], Yi-Shan Tsai [1,3] Hsing-Han Lu [1], Si-Yun Chen [1], Hsin-Ying Hsieh [1] and Chang-Shen Lin [1,4,5,6] *

[1]Graduate Institute of Medicine, College of Medicine, Kaohsiung Medical University, Kaohsiung, Taiwan; ycwang0214@gmail.com (Y.C.W.); 1016ys@gmail.com (Y.S.T.); unrealhank@gmail.com (H.H.L.); qoxoruby@gmail.com (S.Y.C.); hsinying11125@gmail.com (H.Y.H.); csl@kmu.edu.tw (C.S.L.)
[2]Department of Otorhinolaryngology, Kaohsiung Municipal Ta-Tung Hospital, Kaohsiung, Taiwan; kawolee@kmu.edu.tw
[3] Hepatobiliary Division, Department of Internal Medicine, Kaohsiung Medical University Hospital, Kaohsiung Medical University, Kaohsiung, Taiwan
[4]Center for Cancer Research, Kaohsiung Medical University, Kaohsiung, Taiwan
[5]Department of Medical Research, Kaohsiung Medical University Hospital, Kaohsiung Medical University, Kaohsiung, Taiwan
[6]Department of Biological Sciences, National Sun Yat-sen University, Kaohsiung, Taiwan
*****Correspondence: csl@kmu.edu.tw
[†]These authors contributed equally.

Table S1. Patient demographics

		Subsite		
	Total	Larynx	Pharynx	Oral cavity
Variable	100	36	33	31
Male	94	33	31	30
Female	6	3	2	1
Age/yr [a]	21-82	45-82 (64)	41-77 (55)	21-71 (50)
T1	28	13	4	11
T2	30	8	9	13
T3	21	7	11	3
T4	21	8	9	4
N0	63	31	8	24
N1	17	4	9	4
N2	14	0	13	1
N3	6	1	3	2
Dead	47	18	22	7
Alive	53	18	11	24
Follow-up/month [a]	1-86 (26)	6-86 (48)	1-86 (22)	1-49 (15)

[a] In parentheses: median

Table S2 Significant enriched pathways. The top 20 (hallmark), 30 (GO), and 50 (curated) enriched pathways in KU55933-treated HEp-2 cells were shown (corresponding to Figure 5).

NAME	SIZE	ES	NES	NOM p-val	FDR q-val
HALLMARK_INTERFERON_ALPHA_RESPONSE	94	0.652478	2.473751	0	0
HALLMARK_INTERFERON_GAMMA_RESPONSE	193	0.503014	2.133604	0	0
HALLMARK_CHOLESTEROL_HOMEOSTASIS	72	0.532551	1.925983	0	0
HALLMARK_FATTY_ACID_METABOLISM	154	0.429494	1.819194	0	3.26E-04
HALLMARK_P53_PATHWAY	190	0.423794	1.795713	0	5.77E-04
HALLMARK_APOPTOSIS	156	0.400233	1.647806	0	0.0088228
HALLMARK_PEROXISOME	104	0.415096	1.632662	0	0.0082543
HALLMARK_PROTEIN_SECRETION	94	0.408663	1.564826	0.00719425	0.0187001
HALLMARK_UNFOLDED_PROTEIN_RESPONSE	105	0.397442	1.538685	0.00515464	0.0198415
HALLMARK_ADIPOGENESIS	192	0.360381	1.532619	0	0.0187995
HALLMARK_XENOBIOTIC_METABOLISM	197	0.359608	1.519082	0	0.0195834
HALLMARK_UV_RESPONSE_DN	138	0.364522	1.491888	0.00252525	0.023029
HALLMARK_TNFA_SIGNALING_VIA_NFKB	197	0.343746	1.459509	0	0.0274573
HALLMARK_BILE_ACID_METABOLISM	111	0.351424	1.383979	0.01851852	0.0520344
HALLMARK_MTORC1_SIGNALING	193	0.31717	1.359917	0.00943396	0.0620071
HALLMARK_OXIDATIVE_PHOSPHORYLATION	183	0.315418	1.317818	0.02052786	0.0836244
HALLMARK_HEME_METABOLISM	190	0.306543	1.308603	0.01744186	0.0862376
HALLMARK_MYOGENESIS	197	0.299298	1.288095	0.04143646	0.0972822
HALLMARK_ESTROGEN_RESPONSE_EARLY	194	0.292591	1.25122	0.04347826	0.1257918

NAME	SIZE	ES	NES	NOM p-val	FDR q-val
GO_NEGATIVE_REGULATION_OF_VIRAL_GENOME_REPLICATION	55	0.612991	2.156	0	0.00424634
GO_AUTOPHAGOSOME	90	0.542889	2.063988	0	0.01799957
GO_AMINE_CATABOLIC_PROCESS	21	0.71793	2.027533	0	0.02683295
GO_REGULATION_OF_VIRAL_GENOME_REPLICATION	89	0.520876	1.996419	0	0.03571902
GO_ENDOSOME_ORGANIZATION	73	0.533415	1.969465	0	0.04821258
GO_AUTOPHAGOSOME_ORGANIZATION	91	0.510365	1.967323	0	0.0413958
GO_REGULATION_OF_CHOLESTEROL_METABOLIC_PROCESS	55	0.56386	1.963015	0	0.03850474
GO_VESICLE_TARGETING_TO_FROM_OR_WITHIN_GOLGI	73	0.546246	1.960321	0	0.03501321
GO_SHORT_CHAIN_FATTY_ACID_METABOLIC_PROCESS	16	0.761413	1.956721	0	0.03310475
GO_COPII_COATED_VESICLE_BUDDING	71	0.529213	1.955539	0	0.03052714
GO_NEGATIVE_REGULATION_OF_VIRAL_LIFE_CYCLE	78	0.529684	1.948738	0	0.03196811
GO_GOLGI_VESICLE_BUDDING	79	0.522019	1.946524	0	0.03018545
GO_NEGATIVE_REGULATION_OF_TYPE_I_INTERFERON_PRODUCTION	42	0.598382	1.944613	0	0.02883971
GO_ACETYL_COA_METABOLIC_PROCESS	38	0.608086	1.938715	0	0.02979784
GO_INTRINSIC_APOPTOTIC_SIGNALING_PATHWAY_IN_RESPONSE_TO_ENDOPLASMIC_RETICULUM_STRESS	57	0.554325	1.937517	0	0.0283755
GO_NEGATIVE_REGULATION_OF_EXECUTION_PHASE_OF_APOPTOSIS	20	0.689639	1.918355	0	0.03596581
GO_NEGATIVE_REGULATION_OF_VIRAL_PROCESS	94	0.491981	1.906699	0	0.04086911
GO_GLUTATHIONE_METABOLIC_PROCESS	46	0.571256	1.905026	0	0.03942038
GO_SNAP_RECEPTOR_ACTIVITY	31	0.610648	1.903475	0	0.03806338
GO_VESICLE_TARGETING	93	0.502253	1.902141	0	0.03674332
GO_INTRA_GOLGI_VESICLE_MEDIATED_TRANSPORT	31	0.609275	1.897203	0.00233645	0.03764508
GO_REGULATION_OF_EXECUTION_PHASE_OF_APOPTOSIS	36	0.591921	1.89466	0	0.03795059
GO_REGULATION_OF_VACUOLE_ORGANIZATION	43	0.556485	1.8939	0	0.0368506
GO_FATTY_ACID_CATABOLIC_PROCESS	102	0.483186	1.893501	0	0.03553407
GO_CELLULAR_PIGMENTATION	50	0.559204	1.893151	0	0.03440539
GO_AUTOPHAGOSOME_MEMBRANE	34	0.606495	1.892333	0	0.03352436
GO_SELECTIVE_AUTOPHAGY	44	0.564285	1.887347	0	0.03489736
GO_CYTOPLASMIC_PATTERN_RECOGNITION_RECEPTOR_SIGNALING_PATHWAY_IN_RESPONSE_TO_VIRUS	23	0.669233	1.880908	0	0.03644766
GO_NUCLEOTIDE_SUGAR_METABOLIC_PROCESS	37	0.581965	1.870798	0	0.04035633
GO_PEROXISOME_ORGANIZATION	78	0.501758	1.869793	0	0.03946466

Table S2 (cont.) Significant enriched pathways. The top 20 (hallmark), 30 (GO), and 50 (curated) enriched pathways in KU55933-treated HEp-2 cells were shown (corresponding to Figure 5).

NAME	SIZE	ES	NES	NOM p-val	FDR q-val
ONDER_CDH1_TARGETS_1_UP	133	0.639405	2.611252	0	0
MOSERLE_IFNA_RESPONSE	31	0.822262	2.49187	0	0
BROWNE_INTERFERON_RESPONSIVE_GENES	65	0.685776	2.483403	0	0
BOSCO_INTERFERON_INDUCED_ANTIVIRAL_MODULE	74	0.663132	2.454022	0	0
DAUER_STAT3_TARGETS_DN	46	0.72371	2.393067	0	0
BLUM_RESPONSE_TO_SALIRASIB_UP	227	0.549361	2.383227	0	0
KOBAYASHI_EGFR_SIGNALING_24HR_UP	90	0.613625	2.297045	0	1.88E-04
PODAR_RESPONSE_TO_ADAPHOSTIN_UP	134	0.560341	2.288584	0	1.64E-04
STAMBOLSKY_TARGETS_OF_MUTATED_TP53_DN	46	0.669546	2.280742	0	2.93E-04
JI_RESPONSE_TO_FSH_UP	61	0.645536	2.267136	0	2.64E-04
ZHANG_INTERFERON_RESPONSE	23	0.791072	2.250403	0	2.40E-04
BOWIE_RESPONSE_TO_EXTRACELLULAR_MATRIX	17	0.846095	2.245031	0	2.20E-04
YAN_ESCAPE_FROM_ANOIKIS	23	0.790051	2.243104	0	2.03E-04
BOWIE_RESPONSE_TO_TAMOXIFEN	18	0.833794	2.240633	0	1.89E-04
JOHNSTONE_PARVB_TARGETS_3_UP	398	0.486006	2.238075	0	1.76E-04
GARY_CD5_TARGETS_UP	442	0.474079	2.198456	0	3.28E-04
DER_IFN_ALPHA_RESPONSE_UP	72	0.605413	2.193389	0	3.09E-04
SCHMIDT_POR_TARGETS_IN_LIMB_BUD_UP	26	0.738738	2.178879	0	3.66E-04
GAJATE_RESPONSE_TO_TRABECTEDIN_UP	62	0.603531	2.172628	0	3.47E-04
UROSEVIC_RESPONSE_TO_IMIQUIMOD	19	0.800548	2.172549	0	3.29E-04
HORTON_SREBF_TARGETS	23	0.7443	2.17032	0	3.14E-04
LU_EZH2_TARGETS_UP	260	0.489245	2.167492	0	2.99E-04
EINAV_INTERFERON_SIGNATURE_IN_CANCER	26	0.741372	2.14943	0	5.17E-04
GENTILE_UV_HIGH_DOSE_UP	19	0.766134	2.119514	0	6.06E-04
GARCIA_TARGETS_OF_FLI1_AND_DAX1_UP	48	0.630002	2.112917	0	6.36E-04
ONDER_CDH1_TARGETS_3_UP	17	0.788932	2.097404	0	9.14E-04
SEITZ_NEOPLASTIC_TRANSFORMATION_BY_8P_DELETION_UP	68	0.57965	2.092811	0	8.80E-04
REACTOME_INTERFERON_ALPHA_BETA_SIGNALING	62	0.585031	2.080991	0	0.00103685
REACTOME_RESPONSE_OF_EIF2AK1_HRI_TO_HEME_DEFICIENCY	15	0.79878	2.080064	0	0.0010011
XU_AKT1_TARGETS_6HR	25	0.717133	2.077819	0	0.00101196
KIM_LRRC3B_TARGETS	27	0.698747	2.066231	0	0.00136003
BHATI_G2M_ARREST_BY_2METHOXYESTRADIOL_DN	121	0.51158	2.062747	0	0.00140038
HECKER_IFNB1_TARGETS	92	0.537391	2.061776	0	0.00135795
KEGG_SNARE_INTERACTIONS_IN_VESICULAR_TRANSPORT	38	0.634727	2.059567	0	0.00135727
WANG_RESPONSE_TO_GSK3_INHIBITOR_SB216763_UP	327	0.450018	2.053595	0	0.00146748
KANNAN_TP53_TARGETS_UP	56	0.592377	2.05234	0	0.00146338
TIEN_INTESTINE_PROBIOTICS_24HR_DN	201	0.477765	2.040164	0	0.00180814
LIM_MAMMARY_STEM_CELL_DN	392	0.442893	2.035357	0	0.00193089
DER_IFN_GAMMA_RESPONSE_UP	69	0.563483	2.030513	0	0.00208405
REACTOME_CHOLESTEROL_BIOSYNTHESIS	23	0.71554	2.026511	0	0.00219606
DER_IFN_BETA_RESPONSE_UP	100	0.518834	2.004372	0	0.00316711
MISSIAGLIA_REGULATED_BY_METHYLATION_UP	116	0.509259	2.001158	0	0.00331215
BENNETT_SYSTEMIC_LUPUS_ERYTHEMATOSUS	27	0.665133	2.001061	0	0.00323512
GERHOLD_ADIPOGENESIS_UP	47	0.593377	1.997695	0	0.00337045
MARIADASON_REGULATED_BY_HISTONE_ACETYLATION_UP	53	0.580227	1.991533	0	0.00367342
PACHER_TARGETS_OF_IGF1_AND_IGF2_UP	35	0.631071	1.988656	0	0.00382142
ZHAN_MULTIPLE_MYELOMA_CD1_VS_CD2_UP	63	0.557163	1.984906	0	0.00391025
RADAEVA_RESPONSE_TO_IFNA1_UP	48	0.581426	1.961379	0	0.00533943
HELLER_HDAC_TARGETS_UP	276	0.440686	1.955595	0	0.00584746
SMIRNOV_RESPONSE_TO_IR_6HR_UP	158	0.467222	1.944896	0	0.00693984

Table S3 Significant core enriched genes[a] in the gene sets of HALLMARL_APOPTOSIS, GO_INTRINSIC_APOPTOTIC_SIGNALING_PATHWAY_IN_RESPONSE_TO_ENDOPLASMIC_RETICULUM_STRESS, and KERLEY_RESPONSE_TO_CISPLATIN_UP pathways (corresponding to Figure 5).

HALLMARK_APOPTOSIS	GO_INTRINSIC_APOPTOTIC_SIGNALING_PATHWAY_IN_RESPONSE_TO_ENDOPLASMIC_RETICULUM_STRESS	KERLEY_RESPONSE_TO_CISPLATIN_UP
DDIT3	DDIT3	GDF15
ATF3	BBC3	ACTA2
ISG20	CHAC1	GADD45A
SQSTM1	RNF183	CDKN1A
SAT1	TRIB3	TP53TG1
GADD45A	PMAIP1	ZMAT3
CTH	PML	PLK3
CDKN1A	CASP4	DGKA
CASP1	ATF4	DDB2
RNASEL	CEBPB	BTG2
PMAIP1	XBP1	PLAT
IL1A	OPA1	ANXA4
SOD2	TP53	ALDH1A3
CASP4	BAX	RPS27L
GADD45B	ERP29	TNFRSF10C
BTG2	EIF2AK3	
PLAT	HERPUD1	
GPX4	PTPN2	
TAP1	PPP1R15A	
PEA15	CREB3L1	
PDCD4	DAB2IP	
MGMT	NCK1	
IRF1	TXNDC12	
MADD	TMEM117	
CYLD	BAG6	
EBP		
XIAP		
PDGFRB		
RETSAT		
DAP		
IL1B		
SLC20A1		
BIRC3		
CDC25B		
GCH1		
CFLAR		
BMF		
TIMP2		
HMOX1		
BAX		
GPX1		
TIMP1		
DNM1L		
CASP8		
JUN		
FDXR		
GSR		
IL6		
IL18		

[a] The subset of genes that contributes most to the enrichment result within the gene set. (https://www.gsea-msigdb.org/gsea/doc/GSEAUserGuideFrame.html)

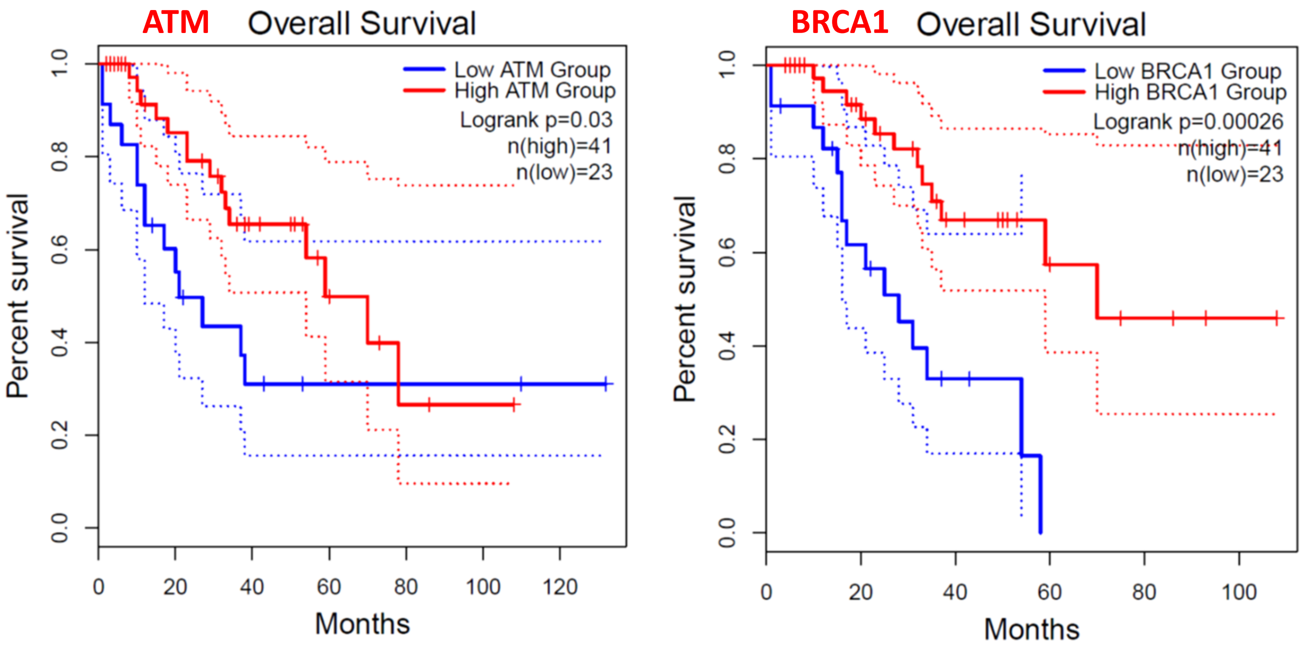

Figure S1. Kaplan-Meier analysis of overall survival (OS) in the atypical and classic molecular subtypes in TCGA HNSC cohort. (corresponding to Figure 3).

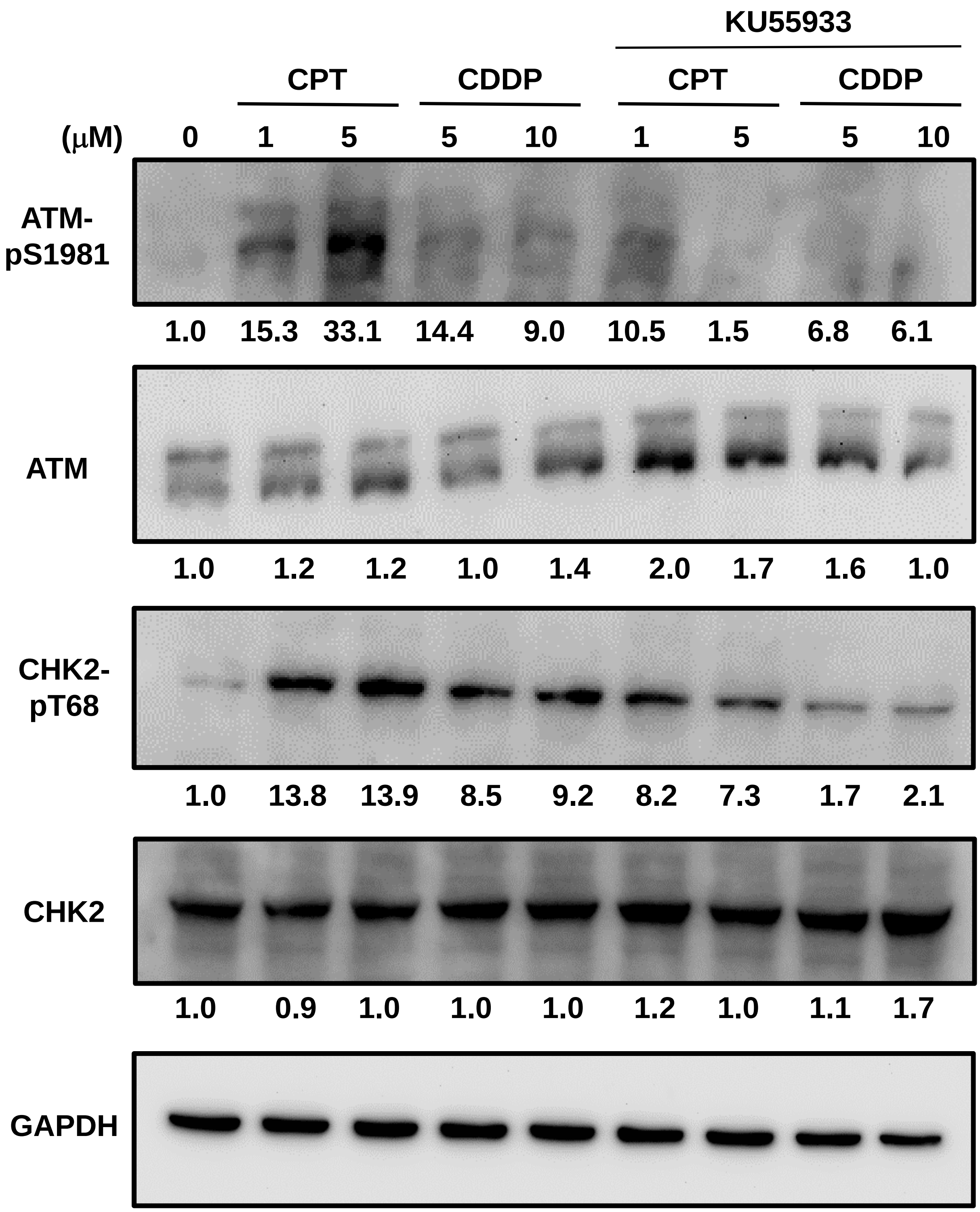

Figure S2. The ATM kinase inhibitor KU55933 inhibits camptothecin (CPT)- and cisplatin (CDDP)-induced ATM signaling in HEp-2 cells. (corresponding to Figures 5, 6). Western blot analysis of ATM and CHK2 (normalized by GAPDH) and their phosphorylated active forms (normalized by total forms) in HEp-2 cells treated with CPT or CDDP with and without KU55933 (20 μM, 3 h). The full-length blots can be found at Figure S7.

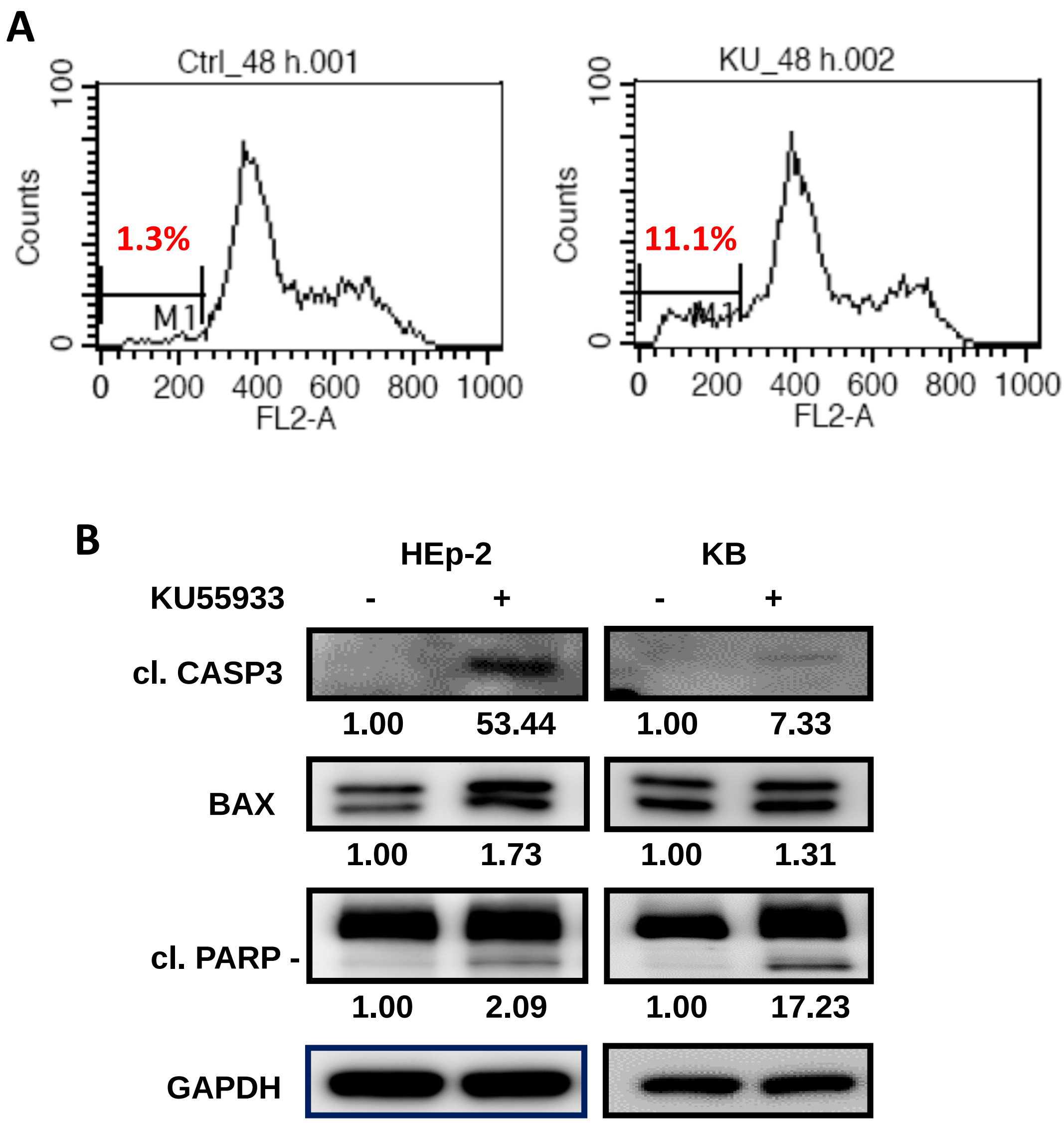

Figure S3. The ATM kinase inhibitor KU55933 induces apoptosis in cancer cells. (corresponding to Figures 5, 6) (**A**) Flow cytometric analysis for propidium iodide-staining of KU55933-treated HEp-2 cells (48 h). Ctrl, vehicle control. KU, KU55933. (**B**) Western blot analysis of BAX and cleaved (cl.) caspase 3 and PARP in KU55933-treated HEp-2 and KB cells (48 h). The full-length blots can be found at Figure S7.

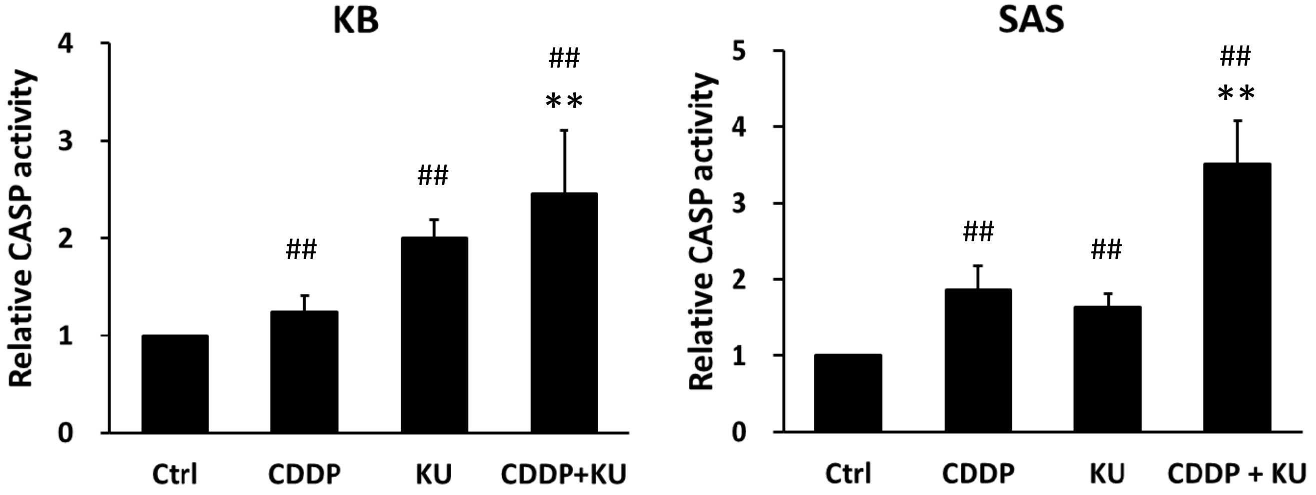

Figure S4. Caspase 3/7 activity is increased by the combination treatment of cisplatin (5 μM) and KU55933 (20 μM) for 24 h in KB and SAS cells. (corresponding to Figures 6). All data are shown as mean ± standard deviation (n = 3). Ctrl, DMSO vehicle control. CDDP, cisplatin. KU, KU55933. **, *P* < 0.01 versus CDDP. ##, *P* < 0.01 versus DMSO.

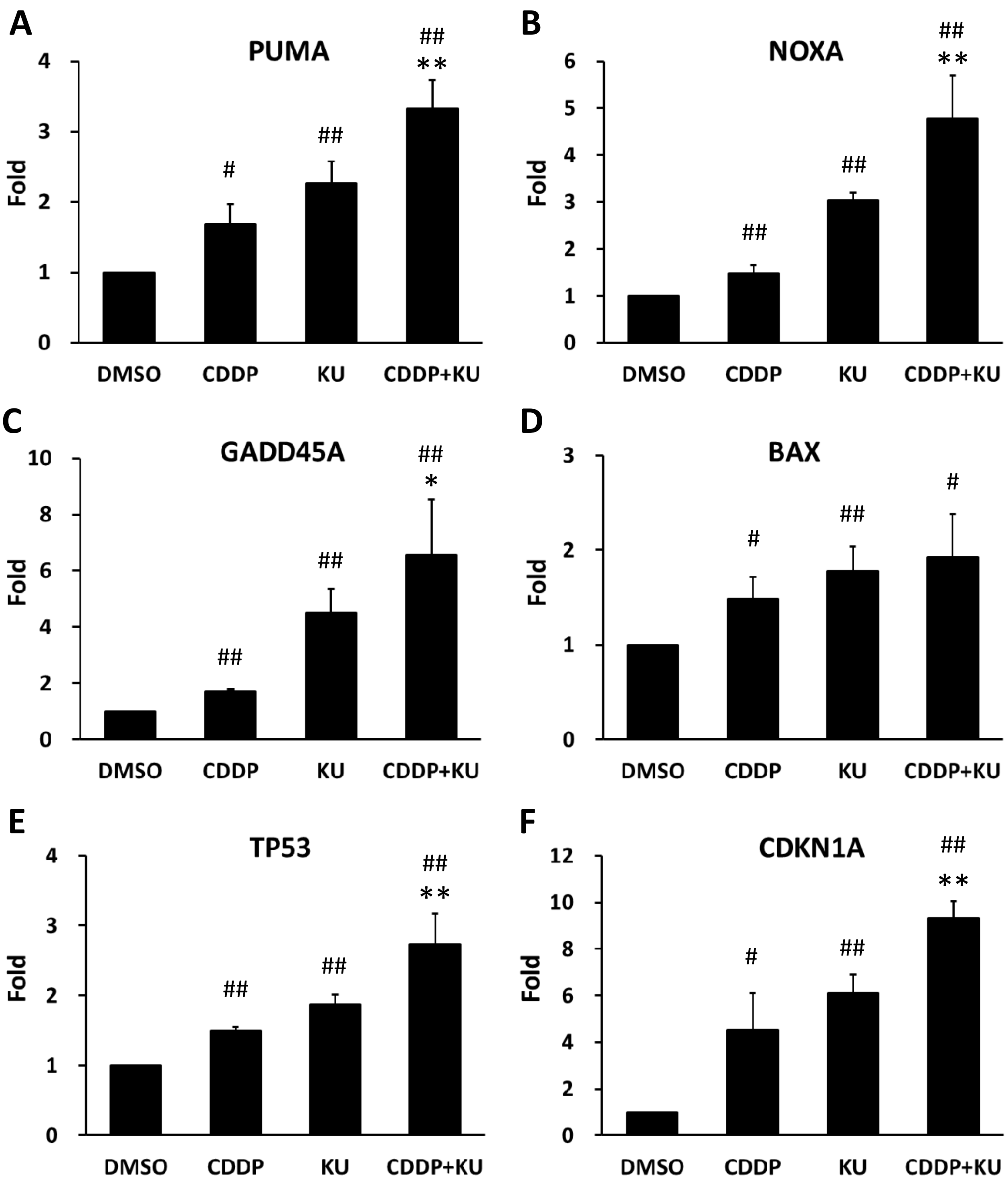

Figure S5. The ATM kinase inhibitor KU55933 augments cisplatin-induced apoptosis gene expression in KB cells (corresponding to Figure 6). RT-qPCR analysis for the gene expression of PUMA (**A**), NOXA (**B**), GADD45A (**C**), BAX (**D**), TP53 (**E**), and CDKN1A (**F**) after treatments of cisplatin (5 μM) and KU55933 (10 μM), either alone or in combination, for 48 h. All data are shown as mean ± standard deviation (n = 3). DMSO, vehicle control. CDDP, cisplatin. KU, KU55933.*, $P < 0.05$ versus CDDP. **, $P < 0.01$ versus CDDP. #, $P < 0.05$ versus DMSO. ##, $P < 0.01$ versus DMSO.

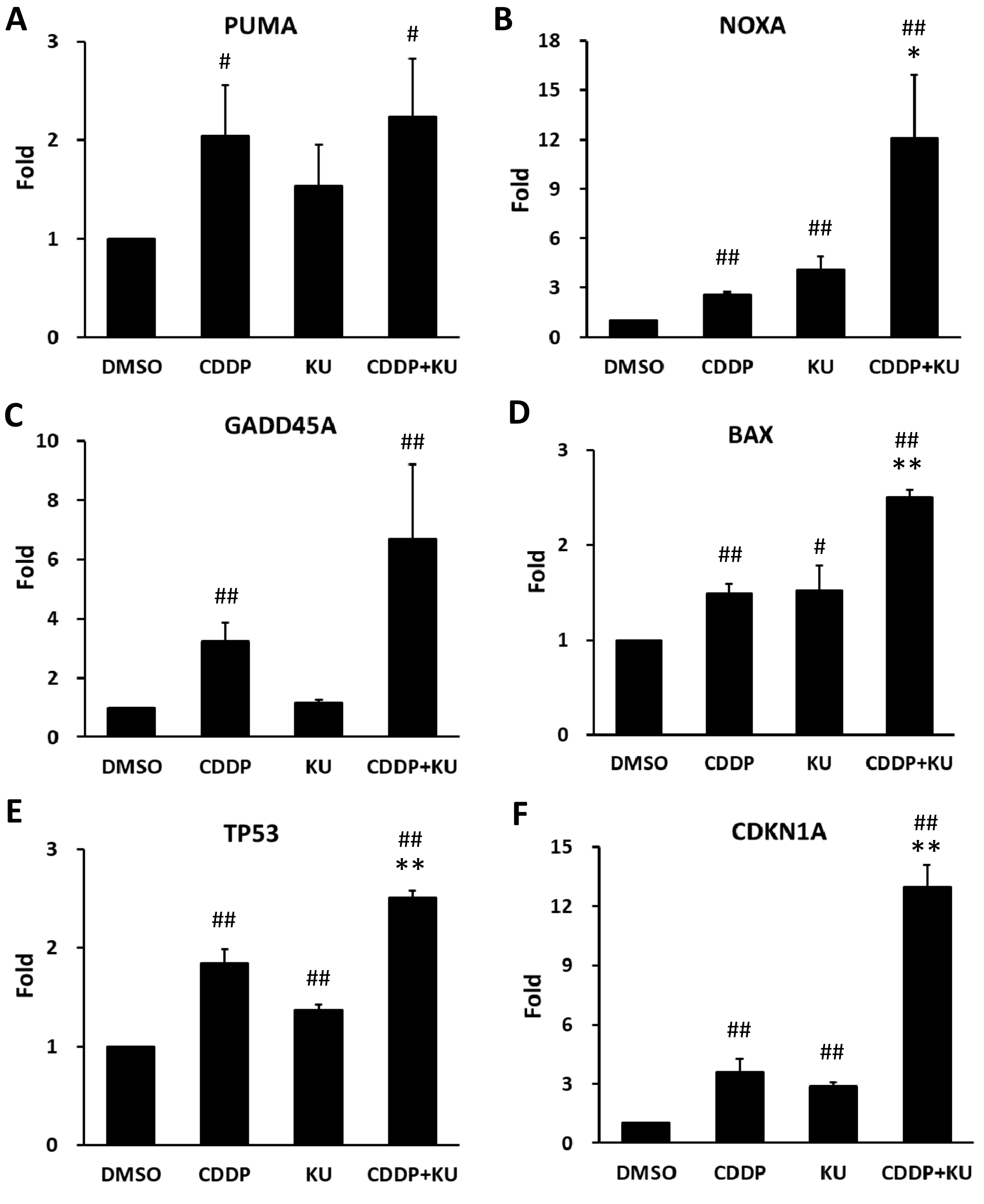

Figure S6. The ATM kinase inhibitor KU55933 augments cisplatin-induced apoptosis gene expression in SAS cells (corresponding to Figure 6). RT-qPCR analysis for the gene expression of PUMA (**A**), NOXA (**B**), GADD45A (**C**), BAX (**D**), TP53 (**E**), and CDKN1A (**F**) after treatments of cisplatin (5 μM) and KU55933 (10 μM), either alone or in combination, for 48 h. All data are shown as mean ± standard deviation (n = 3). DMSO, vehicle control. CDDP, cisplatin. KU, KU55933.*, *P* < 0.05 versus CDDP. **, *P* < 0.01 versus CDDP. #, *P* < 0.05 versus DMSO. ##, *P* < 0.01 versus DMSO.

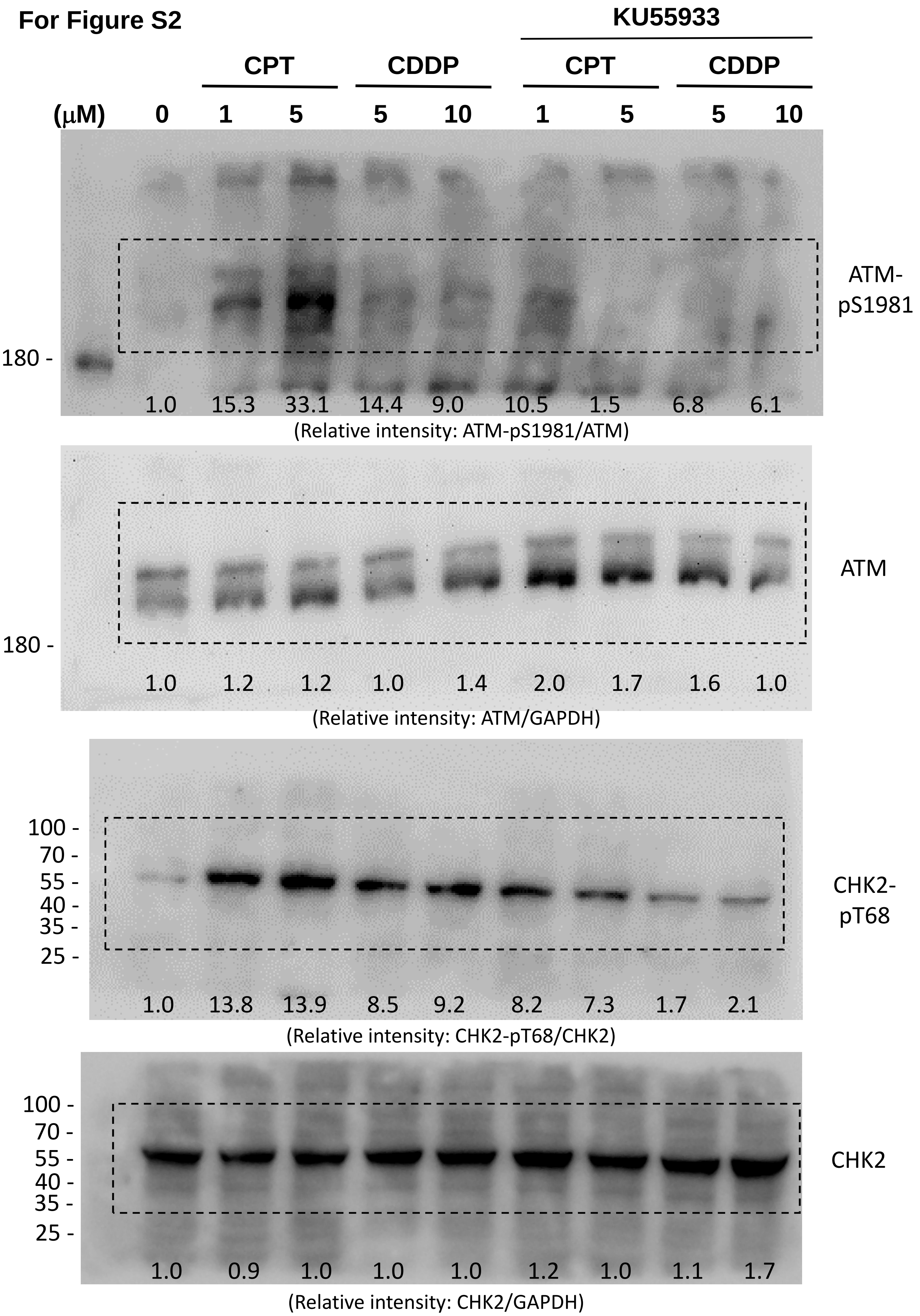

Figure S7. The full-length blots for Supplementary Figure S2 and S3B.

For Figure S2

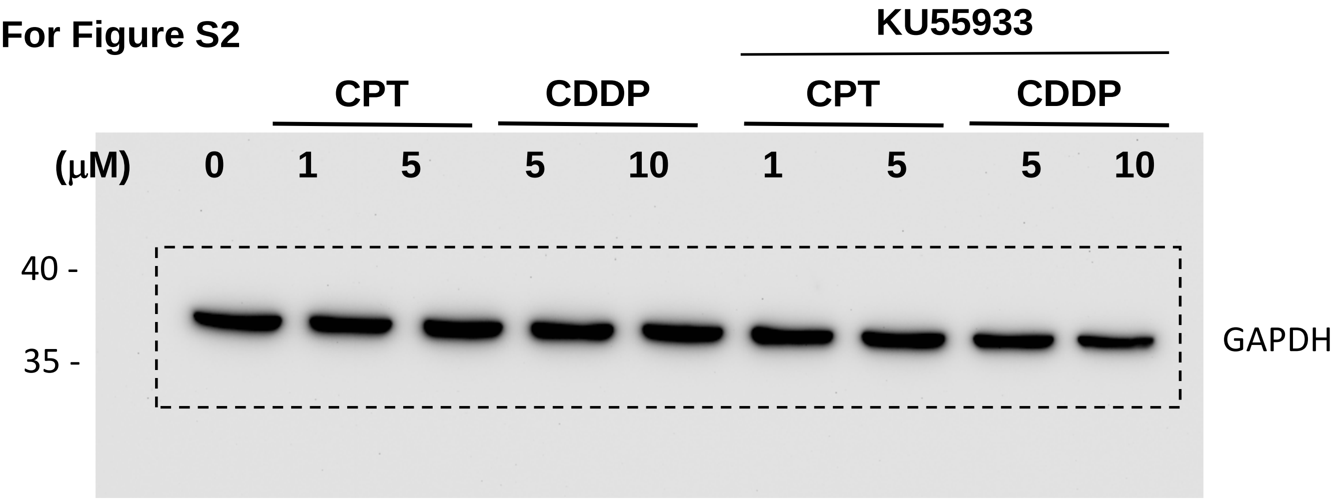

For Figure S3

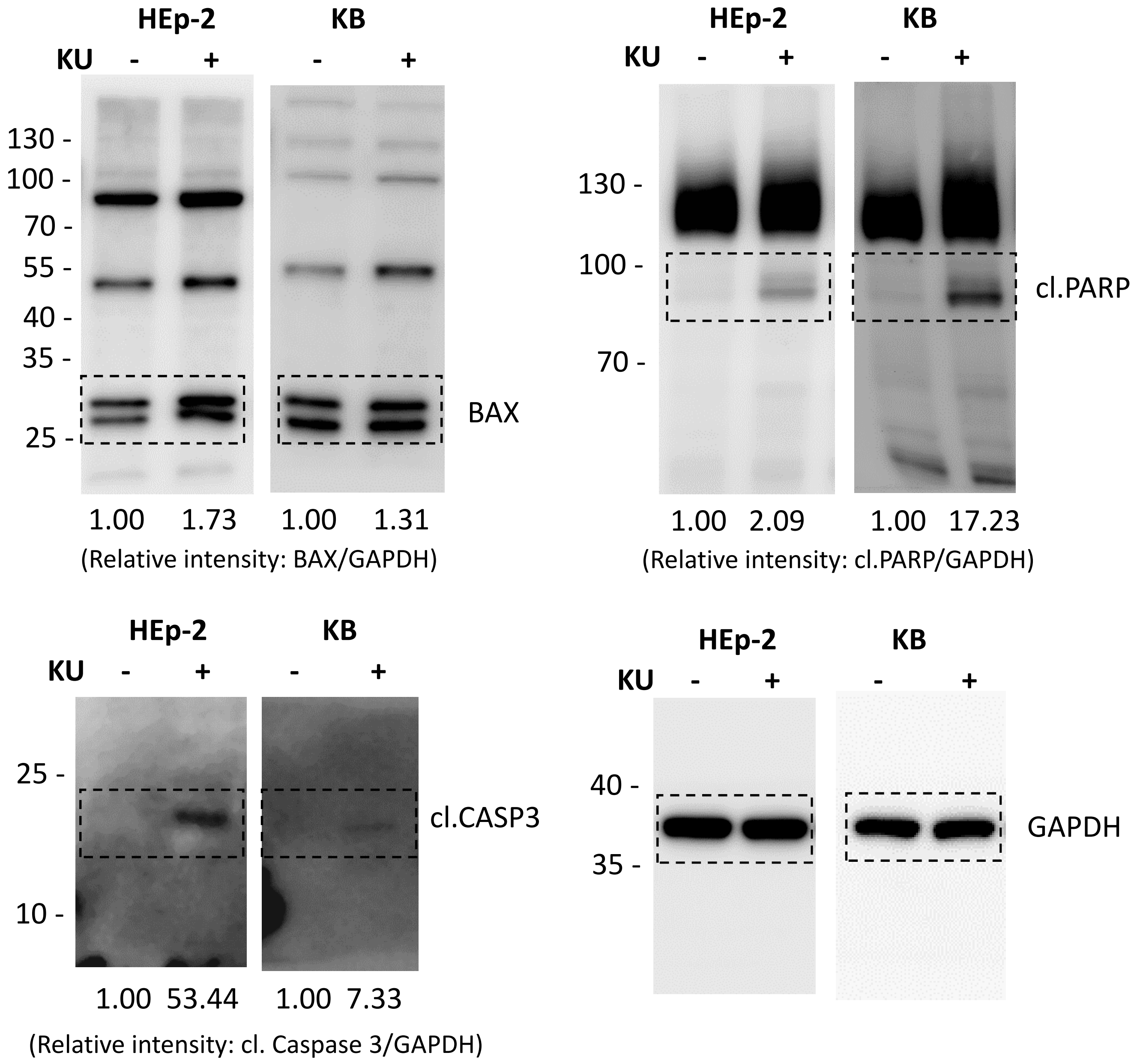

Figure S7 (cont.). The full-length blots for Supplementary Figure S2 and S3B.
